# The Azores plume origin of the New England Seamounts chain

**DOI:** 10.1126/sciadv.aee1780

**Published:** 2026-11-13

**Authors:** Mengxue Liu, Shijie Xie, Dinghui Yang, Taras Gerya, Lijun Liu, Rui Qi, Xinjian Shan, Xingpeng Dong, Xiaobing Xu

**Affiliations:** ^1^ State Key Laboratory of Earthquake Dynamics, Institute of Geology, China Earthquake Administration, Beijing 100029, China.; ^2^ Department of Mathematical Sciences, Tsinghua University, Beijing 100084, China.; ^3^ Institute of Geophysics, Department of Earth and Planetary Sciences, ETH Zurich, Zurich, Switzerland.; ^4^ State Key Laboratory of Lithospheric Evolution, Institute of Geology and Geophysics, Chinese Academy of Sciences, Beijing 100029, China.

## Abstract

Age-progressive seamount chains are fundamental archives in revealing the past plate motion and mantle convection. However, for a representative example such as the New England Seamounts chain on the North American Plate, both its plume source and its evolutionary trajectory remain debatable. This long-lasting uncertainty is mainly caused by the absence of quantitative high-resolution constraints on plume dynamics and its interactions with multiple plates. Here, we aim to answer this question using data-assimilation–based numerical simulations. We propose that the New England chain and the Corner Seamounts on the North American Plate, the Great Meteor Seamounts on the African Plate, and the Azores archipelago on the Eurasian Plate were created by a single plume—the Azores plume. This plume experienced two stages of motion through time. Subduction-induced sublithospheric mantle flow exerts a dominant control on the spatiotemporal distribution of volcanoes along the track and the geometry of this track, surpassing the influence of plume-plate interaction. The latter primarily affects the continuity of this trans-plate hotspot system. These findings offer a reinterpretation of hotspot-track formation and new constraints on how mantle flow reorganizes across multiple plates.

## INTRODUCTION

Seamount chains and hotspot tracks characterized by linear-age progression are widely interpreted as the surface expressions of mantle plumes (*1*, *2*). Typically, the arrival of an ascending plume delivers excess thermal energy into the upper mantle, triggering enhanced magmatism (*1*, *3*). When there is relative motion between the lithospheric plate and the plume, linear chains of volcanic islands and seamounts may form, as is the well-known case with the Hawaiian-Emperor chain (*4*). Consequently, the seamount chains preserve critical information about the history of plate motion and mantle convection, which are of great importance to understanding mantle dynamics (*2*–*6*).

The New England Seamounts chain is the long-lived hotspot track in the North Atlantic Ocean, ranging from the upper continental rise (e.g., Monteregian Hills) to the undersea volcanoes (e.g., Nashville seamount) (Fig. 1) (*6*–*8*). However, the question of the location of the plume being responsible for this track, which is fundamental to revealing the evolution of the track and plume-plate interaction, remains debated. At an early stage, a great part of studies supported the conceptual model that the New England Seamounts chain and the Great Meteor Seamounts were hypothesized to be conjugate features formed by a single plume located near the Great Meteor on the African Plate (AFP), as suggested by numerical modeling, plate reconstruction, and geochemical and geochronologic data (*6*–*12*). However, due to uncertainties in analyzed techniques and the scarcity of samples, it remains unclear whether the New England chain includes the Corner Rise Seamounts chain (*9*). Later, Gente *et al.* (*13*) argued that two different hotspots would be responsible for the New England Seamounts chain and the Great Meteor Seamounts, as the basalts sampled along the former have an HIMU trend, while those along the latter have an EM trend. Instead, they suggested that the Great Meteor and the Azores archipelago were both formed by a single plume based on the similarities in their geochemical signatures, crustal structures, and bathymetric features. As a result, they speculated that the plume source lies beneath the Azores (*13*). More recent elemental and isotopic data also reveal a close relationship between the Great Meteor Seamounts and the Azores hotspot (*3*, *13*, *14*). For example, basalts sampled along the Great Meteor Seamounts further exhibit a chemical signature indicating the mixing of HIMU, N-MORB, and the Azores common component (*14*). In short, the main disputes focus on the origin of the New England chain. Proposed hypotheses include an extinct plume once beneath the North American Plate (NAP), the plume beneath the Great Meteor Seamounts, or the plume underneath the Azores archipelago. It is also unclear whether the New England chain is linked to the Corner Seamounts.

**Fig. 1.**
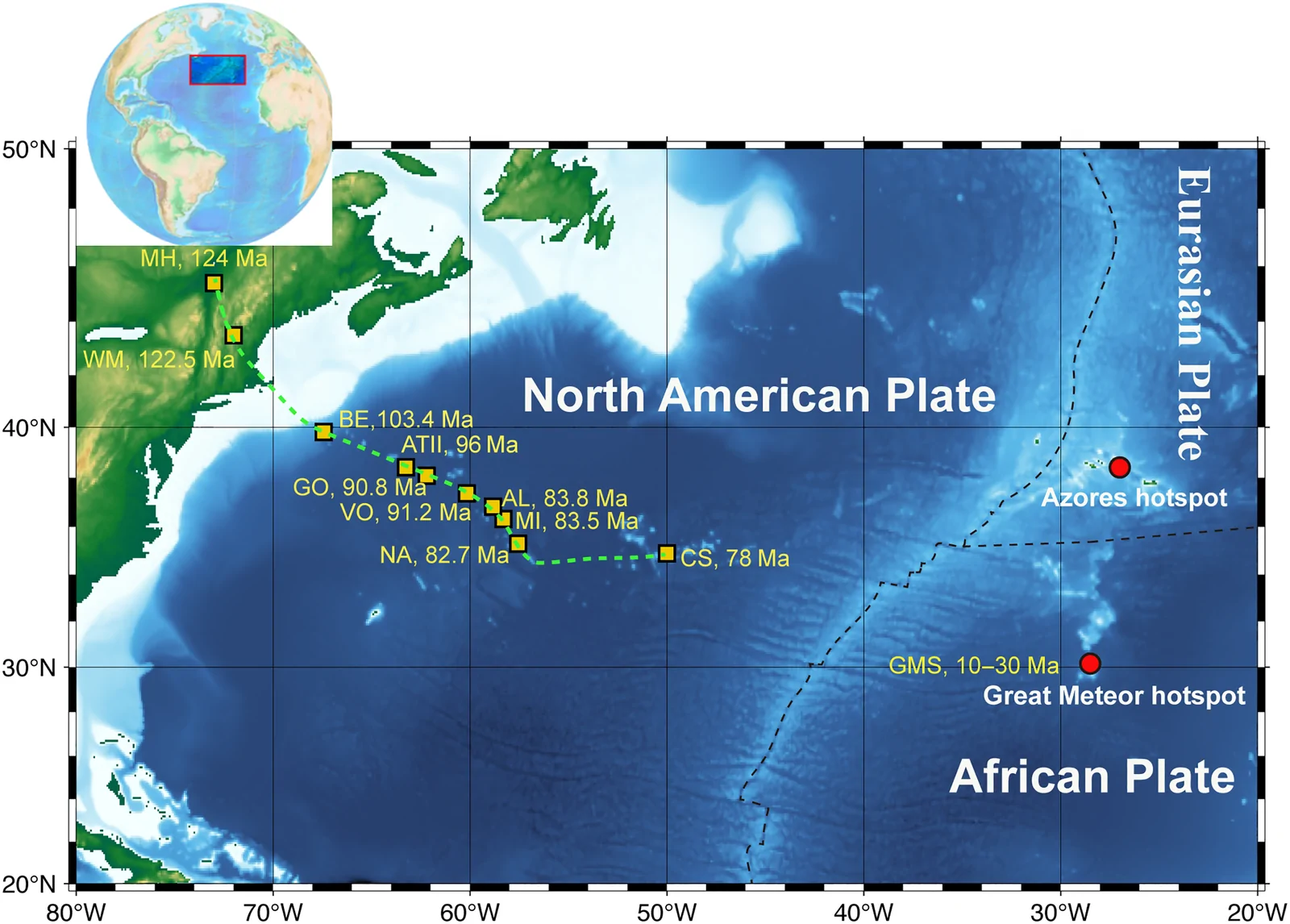
The bathymetric map around the New England Seamounts chain and the Azores hotspot. The hotspot track is outlined by the green dashed line. Seamounts with age data are shown in yellow squares, with the name and age of each seamount beside (*9*, *12*, *15*, *66*–*69*). NH, Monteregian Hills; WM, White Mountains; BE, Bear; ATII, Atlantis II; GO, Gosnold; VO, Vogel; AL, Allegheny; MI, Michael; NA, Nashville; CS, Corner Seamounts; GMS, Great Meteor Seamounts. The locations of the Great Meteor hotspot and Azores hotspot are shown in solid red circles. Black dashed lines are plate boundaries.

Seismic tomography is an efficient way to provide direct evidence of the Earth’s interior structure. Elongate low-velocity bodies are typically interpreted to be hot mantle plumes. However, neither global nor regional imaging has yet detected an upper mantle plume beneath the New England Seamounts or the Great Meteor Seamounts to date (*15*–*18*). In contrast, a clear thermal anomaly has been identified beneath the Azores hotspot (*19*), to the north of the Great Meteor Seamounts, very close to the hotspot inferred by others (*20*). Despite the resolution inconsistencies across different imaging results, this anomaly may at least indicate the presence of an upwelling plume extending to a depth of 150 to 300 km (*21*–*26*).

In summary, multidisciplinary evidence suggests a potential connection between the New England Seamounts chain and the Azores archipelago, indicating that the plume may be beneath the Azores archipelago. Here, we present a series of three-dimensional (3D) thermomechanical numerical experiments to determine the plume source of the New England Seamounts chain and to reconstruct the evolutionary path of the chain. Furthermore, by varying the movement of plume source at 600 km, we explore how plume kinematics influence the plume evolution and the resulting hotspot track. Our model results will gain new insights into the origin and evolution of the New England Seamounts chain, further advancing our understanding of the dynamics of mantle plumes in the presence of moving plates, ridges, and subduction zones.

## RESULTS

All the models we constructed are implemented using the finite element code CitcomS (*27*–*29*) based on our data-assimilation–based numerical method (*30*) (see table S2 in the Supplementary Materials for the model list). The computational domain is large enough to capture the subduction processes within the northeastern Pacific Ocean since the Early Cretaceous. The plume is located nearly in the center of the domain and is far from the model boundaries to avoid the influences of side boundaries (fig. S1). Besides solving thermal-chemical convection, we also consider mantle partial melting and melt source advection to capture more shallow dynamic effects in the upper mantle for the resulting hotspot track. Two different approaches are applied to get surface hotspot tracks (#A and #B, # is the model number) (see Materials and Methods for more details) (*19*, *30*).

In the context, we consider two different plume sources in the models (Materials and Methods). One fixed at 600 km beneath the present-day location of the Great Meteor Seamounts (model 1), and the other fixed underneath the Azores archipelago (model 2) (table S2) (*8*, *19*). Noting that parts of previous studies claimed the Azores plume is not fixed (*17*, *20*, *31*), we also conducted a model with a moving plume source at 600 km. The plume motion and plate motion are described in a deep mantle reference frame. As previous studies (*17*) inferred a northward drift of the Azores plume at ∼1 to 2 cm/year during the past ∼85 million years (Ma). We first adopted a conservative northward plume source migration of 1 cm/year. However, this case did not satisfactorily reproduce the observed spatial distribution and age progression of the New England Seamounts chain. Because the longitudinal component of the Azores plume motion is not well constrained, we therefore additionally tested several different kinetic source-motion scenarios (table S2). According to the results of models 1A (fig. S2) and 1B (Fig. 2A), both predicted hotspot tracks deviate from the observed track; the Corner Seamounts and the Great Meteor Seamounts are out of the simulated melt eruption zone of model 1B (fig. S2). Models 2A (fig. S3) and 2B (Fig. 2A) show even greater deviations between model results and the observations (fig. S3). In contrast, the predictions of model 3B improve a lot. In model 3B, we incorporate a two-stage motion of the Azores plume: First, it moves northeastward and then turns and move northwestward (Fig. 2C). The results of this model best fit the observations in the following aspects (Fig. 2B): The resulting shape of the hotspot track agrees well with the New England Seamounts chain; the seamounts’ ages along the track of the model are also consistent with the geochronologic measurements (Fig. 2); and the modeled Azores plume morphology is coherent with the seismic tomography images (*17*, *18*, *20*–*22*), showing a deflection of the plume conduit toward the Mid-Atlantic Ridge (MAR) (Fig. 3D).

**Fig. 2.**
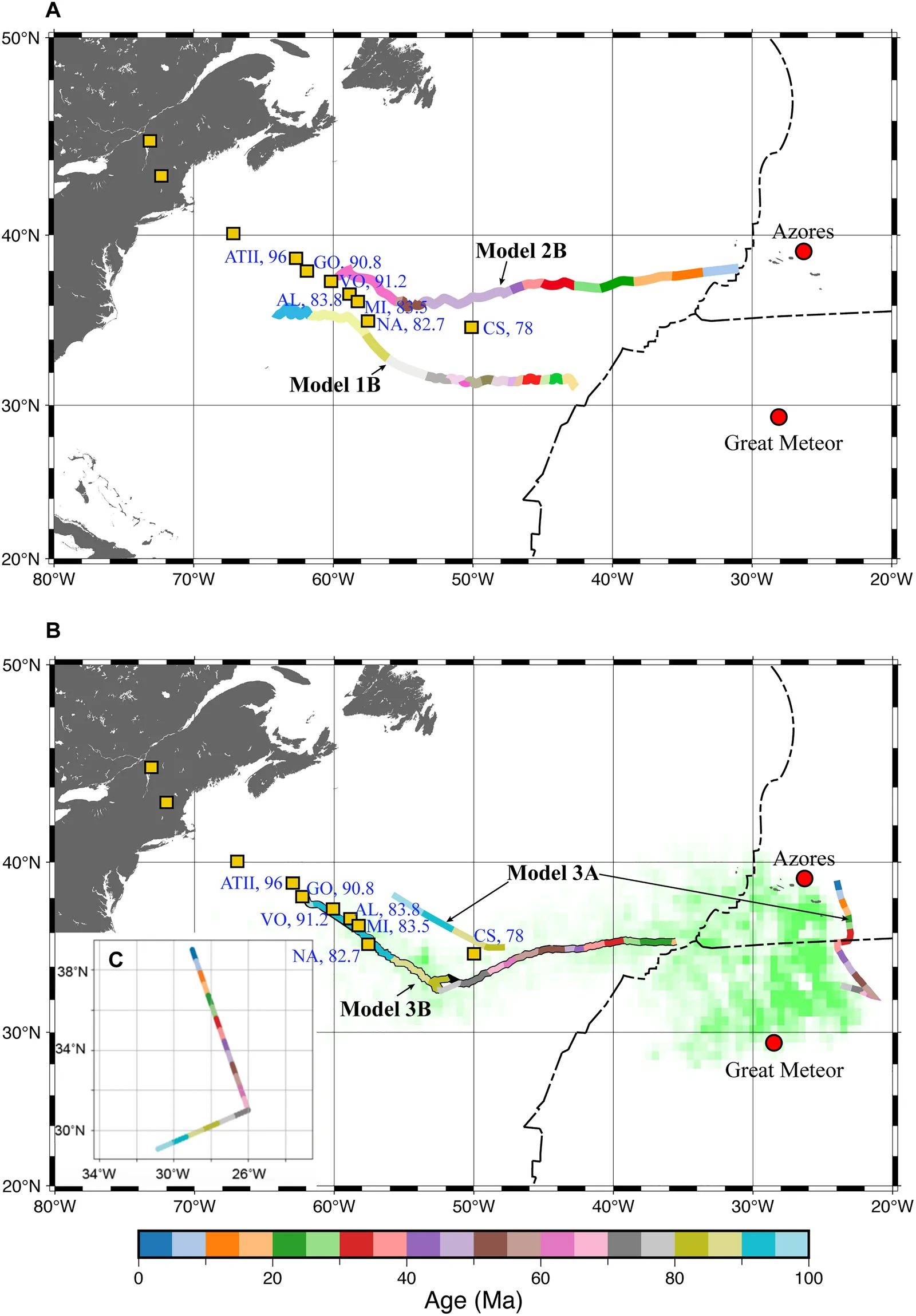
Predicted hotspot tracks of models with different plume source locations. (**A**) Simulated hotspot tracks of models with fixed plumes. In model 1B, the plume source is fixed beneath the Great Meteor Seamounts, and model 2B has a fixed Azores plume. (**B**) Simulated hotspot tracks of models (model 3) with moving Azores plume. Track 3A recording the vertical projection of the plume source onto the moving surface. Track 3B recording the temporally changing location of simulated melt eruption (see Materials and Methods). The green shades are sites of erupted melt predicted by model 3B. The predicted tracks are denoted by colored lines, with the colors showing the ages. The older the age is, the earlier the track forms. The black dashed lines are plate boundaries. The yellow squares show the seamounts, with the blue numbers denoting their names and observed ages. (**C**) Plume source movement used in model 3B (table S2) (*18*).

**Fig. 3.**
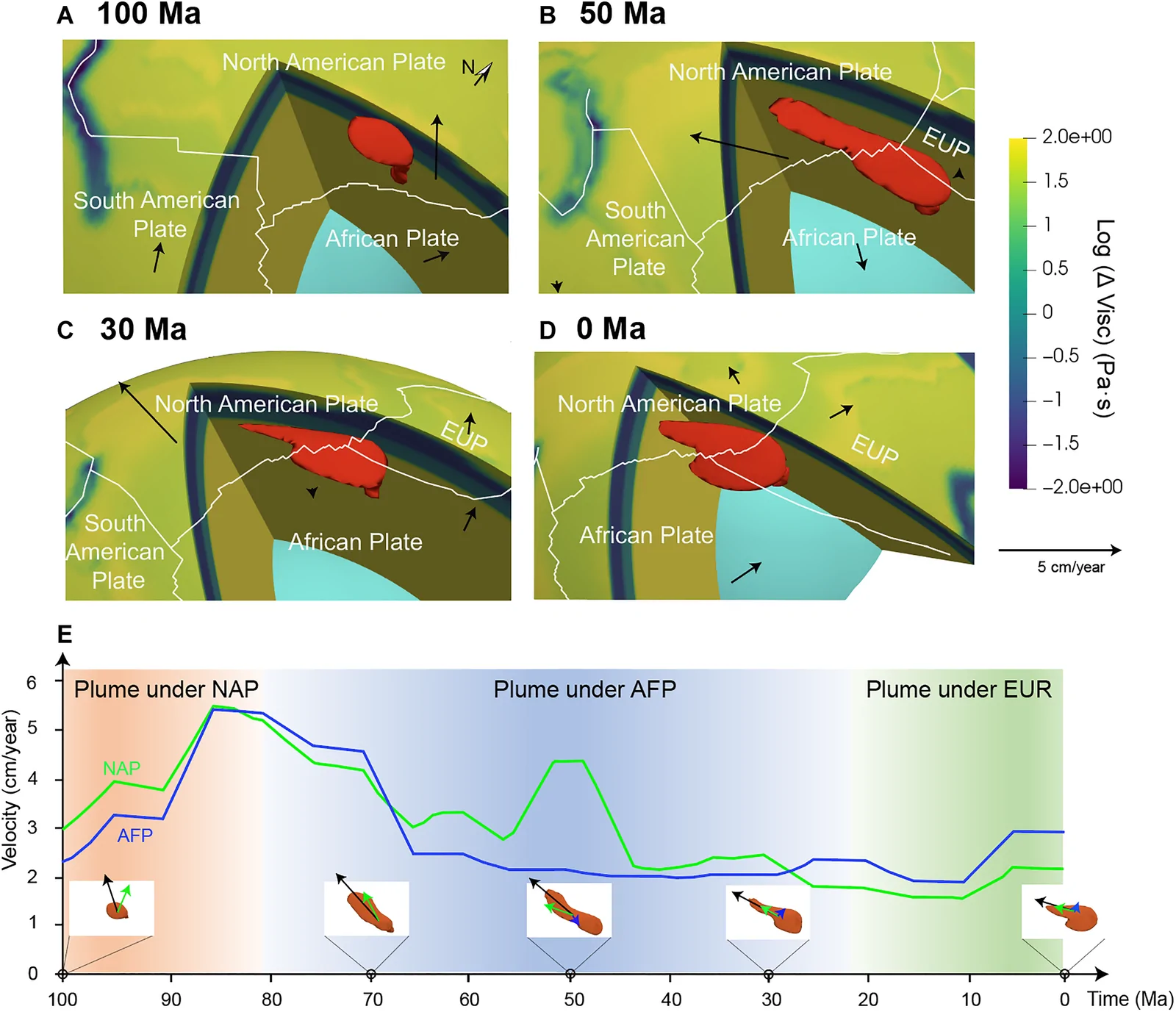
Evolution of model 3B with a moving Azores Plume. (**A**) 100 Ma, (**B**) 50 Ma, (**C**) 30 Ma, and (**D**) 0 Ma. (**E**) shows the velocity magnitude versus time of the NAP and AFP (*34*). EUR, Eurasian Plate. Plumes (red) are denoted by the +100°C temperature anomaly. Insets in (E) show the snapshots of plume morphology at different times; the colored arrows denote the plate motion (orange for NAP, blue for AFP) (*34*) and the mantle flow (black) direction at ∼200-km depth, and the size corresponds to the velocity magnitude. Visc, Viscosity.

According to the reconstruction of model 3B, at the early stage, the plume moves northeastward (Fig. 2C). Before reaching the MAR, the New England (Azores) plume is located beneath the NAP. During this time, the plume conduit shows a pronounced westward deflection that deviates from the direction of the NAP’s motion (Fig. 3A); the New England Seamounts and Corner Seamounts formed on the NAP (Fig. 2). Although the plume approaches and crosses the MAR, its conduit keeps deflecting westward, characterized by an elongated plume head (Fig. 3E). As the model evolves, the plume then turns to move northwestward after ∼70 Ma (Fig. 2C). Under the combined effects of both continuous plume source motion and plate motion, this plume gradually switches from beneath the NAP to beneath the AFP, the Great Meteor Seamounts then formed on the AFP (Figs. 2 and 3, B and E). After that, at ∼10 Ma, the plume experiences another pass-through of a plate boundary (Fig. 3, C to E), the one between the AFP and the Eurasian Plate. The plume eventually arrives at its current location under the Eurasian Plate, ending with the formation of the Azores seamounts group (Figs. 2 and 3D).

Considering the uncertainties in determining the plume motion based on plate reconstruction (*12*, *17*, *18*), we then conducted several additional experiments incorporating different plume motions, categorized into one-phase motion and two-phase motion. Among all the one-phase–motion models, model 4B with a due northward plume motion shows the best fit in the hotspot track. However, its predicted seamount ages deviate from the observations farther compared with model 3B (Figs. 2A and 4, A to C and E to G). Model 7B exhibits very similar predictions to model 3B, with the only significant difference being the seamounts’ ages before ∼90 Ma (Figs. 2A and 4, D and H). To conclude, the model with a two-phase plume motion, initially northeastward before shifting northwestward, provides the most accurate predictions.

**Fig. 4.**
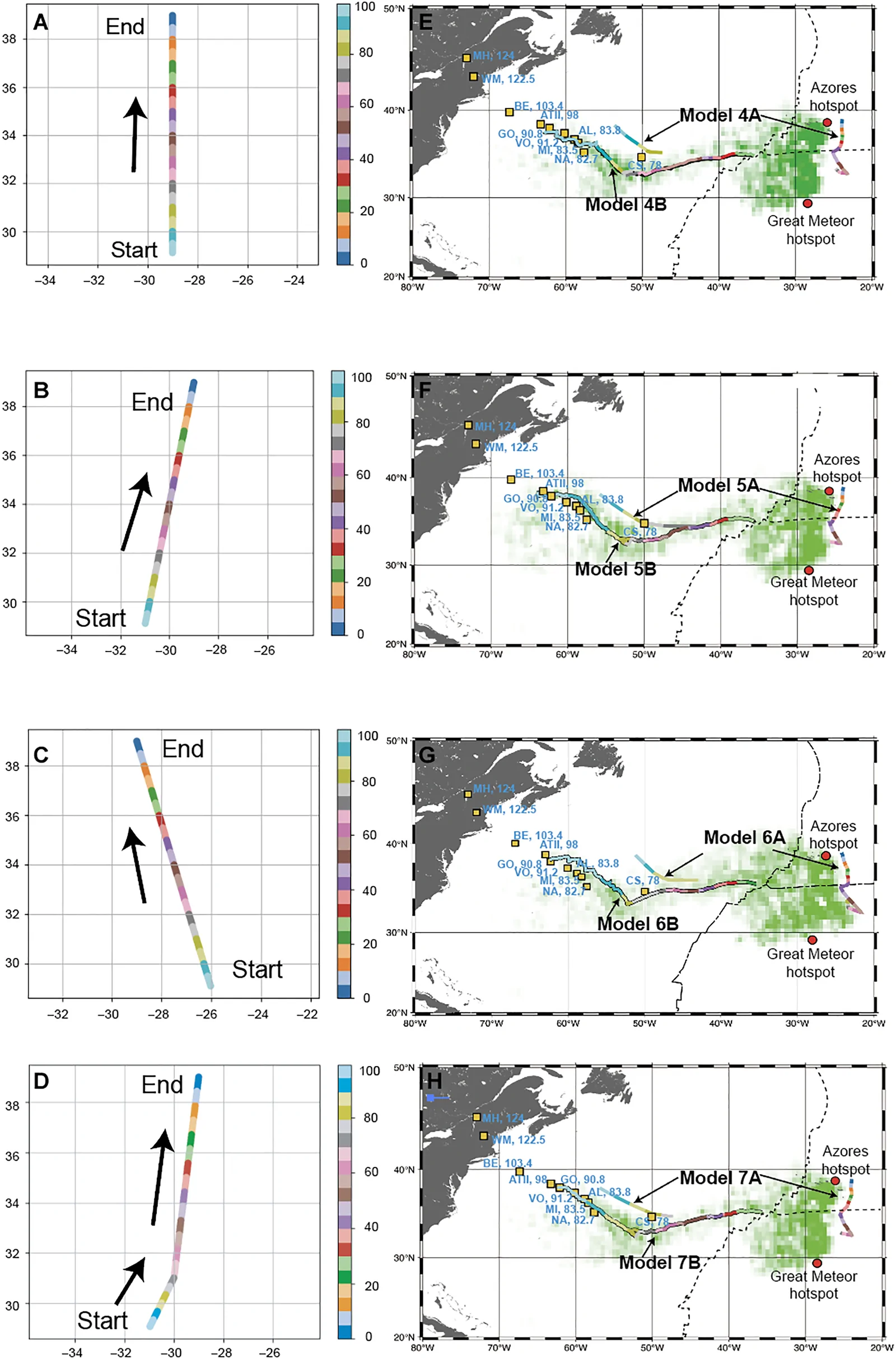
Predicted hotspot tracks of models with different plume source motion. The left panel (**A** to **D**) shows the plume source motion in models (4 to 7), and the right panel (**E** to **H**) shows the corresponding predicted hotspot tracks. Track #A records the vertical projection of the plume source at ∼600-km depth onto the moving surface, and track #B records the temporally changing location of the simulated melt eruption. # is the model number. Annotations of the yellow squares are the same as in Fig. 2.

## DISCUSSION

### Influences of plume motion on the New England–Corner Rise seamounts chain

Although the Corner Rise Seamounts is away from the predicted track in most of our models, it still falls within the melt-eruption zone. We speculate that its formation may be closely linked to the lithospheric structure, consistent with previous studies suggesting that the intraplate magmatism can be fed by a melt channel sourced from a distal plume (*32*). Therefore, we are more supportive of the argument that the New England chain and the Corner Rise Seamounts form a single seamount chain. By comparing the models with different plume motions, we conclude that the starting point of the plume exerts a first-order control on the geographic position of a track, while variations in the plume’s trajectory influence the morphology of the hotspot track. In addition, the change in the predicted orientation of the track at ∼75 to 85 Ma is very similar among these models. During this time, the NAP underwent a significant counterclockwise rotation (from directed northwestward to southwestward) according to plate reconstruction (Figs. 3 and 5) (*33*). This indicates that plume motion at ∼600 km is not the key controlling factor, and the mechanism for this bend seems very different from the case of the Hawaiian-Emperor Bend, which shows that plume motion could contribute a maximum of ∼20° to the bend angle (*30*, *34*), nearly one-third of the total bend. Given that age-progressive seamount chains are widely regarded as important records of the past plate motion (*1*, *2*), we speculate that plate motion variation is more plausibly linked, at least in part, to the bending along the New England chain.

**Fig. 5.**
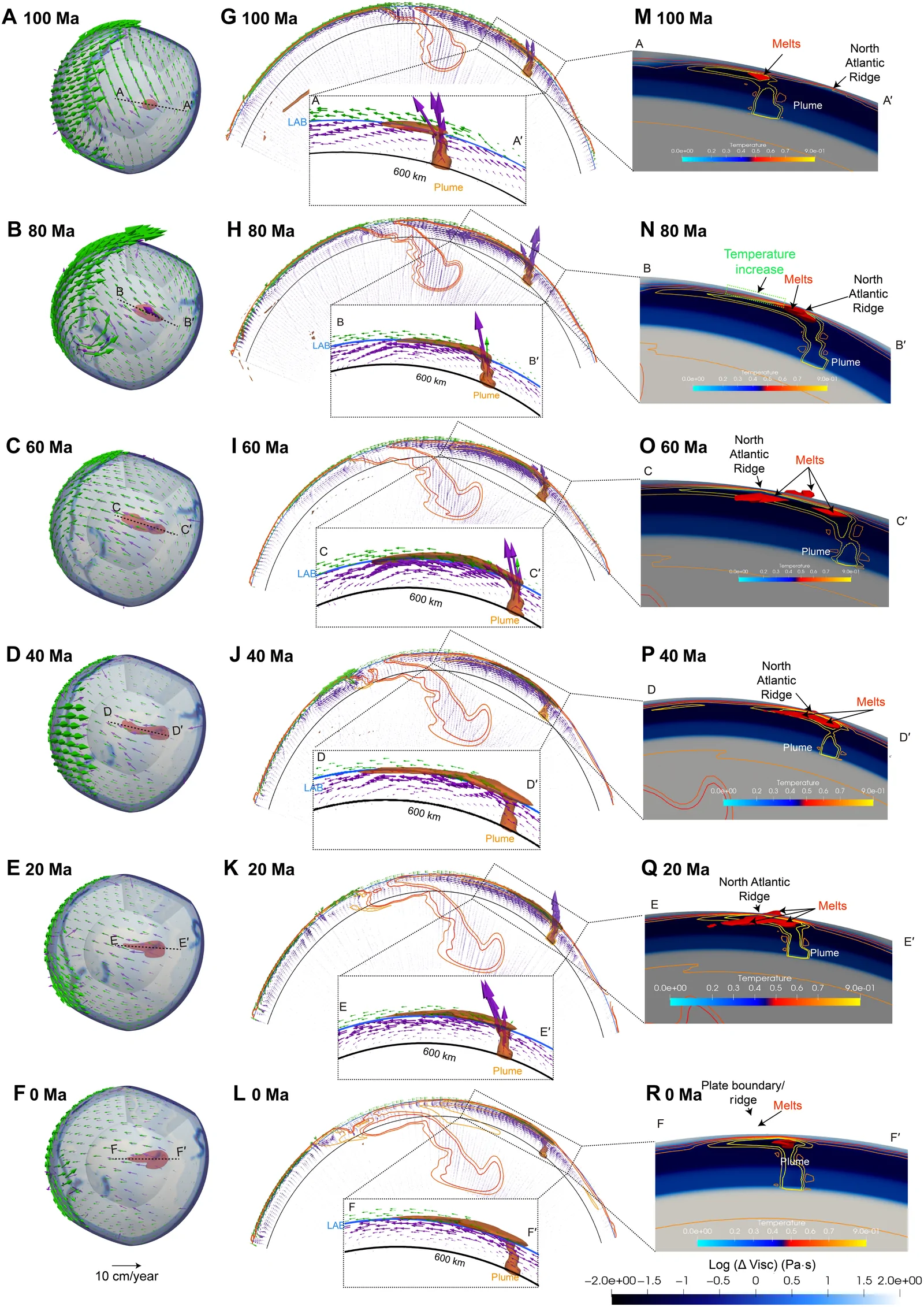
Snapshots of model 3B with a moving Azores Plume. In the left panels (**A** to **F**), green arrows show the predicted velocity at the surface (*V*_surf_), and purple arrows are the velocity in the mantle (*V*_man_; ∼200 km). The size of the arrow is proportional to the velocity magnitude. The orange isosurface represents the plume. It is outlined by a nondimensional temperature contour of ∼0.83 (table S1). AA′-FF′ are the positions of profiles in A2-F2 and A3-F3. The middle panels (**G** to **L**) show the velocity field and the temperature profile across the whole mantle, with the zoomed-in area shown in black dashed rectangular. The green arrows show the predicted velocity in the lithosphere, purple arrows are the velocity in the mantle, and the arrow size is proportional to the velocity magnitude. The blue and black lines are Lithosphere-Asthenosphere Boundary (LAB) and 600-km contours, respectively. The right panels (**M** to **R**) show the snapshots of temperature (isotherms) and viscosity (blue and black background solid color) in the zoomed-in area in A2-F2. The 3D contour of 1% melt fraction is shown in red.

### Missing of the seamounts record between the New England Seamounts and the Great Meteor Seamounts

In this context, we propose that the New England chain, the Great Meteor Seamounts, and the Azores seamounts are formed by a single plume—the Azores plume. If this is the truth, then one of the features of this track is the apparent absence of a clear trace of volcanic edifices or seamounts between the Corner Seamounts and the Great Meteor Seamounts. On the basis of our model predictions, we suggest that this gap may result from the combined effects of subduction-induced sublithospheric mantle flow and plate motion, as well as the plume-ridge interaction.

According to model 3B, this gap is formed during the period when the plate motion (both velocity magnitude and direction) changed significantly (Figs. 3 and 5). Before the plume interacts with the ridge (∼80 Ma), the direction where the plume conduit deviates is mainly controlled by the subduction-induced sublithospheric mantle flow, which points to nearly the opposite side of the MAR. During this time, the elongated plume head significantly heats the above lithosphere, which may facilitate the melt eruption and seamount formation in the interior of the NAP (Fig. 5, M and N). After that, the direction of the NAP motion changed; it gradually shifts to align with the sublithospheric mantle flow. Under the combined effect of plate motion and the subduction-induced mantle flow, a portion of the plume’s hot material rapidly traveled horizontally after reaching the bottom of the lithosphere (Figs. 3 and 5, I and O), which may reduce the accumulation and, consequently, eruption of the melt in situ. The remaining plume material then flows toward the MAR with a thinner lithosphere and advected along the ridge axis. This has been proposed by previous numerical modeling studies: When plume and ridge interact and the ridge spreads slowly, the plume’s hot materials are more inclined to flow along the ridge’s axis (*13*). The above extensive horizontal motions of plume material may intensify its cooling and suppress focused volcanic signatures. Besides, along the ridge axis, the newly erupted materials repeatedly cover the old ones due to the slow spreading of the MAR (Fig. 5, O, P, and Q, and fig. S4), which may also contribute to the missing of clear traces of volcanic edifices on both NAP and AFP. Previous geochemical studies have reported that the basalts sampled from positions near the Corner Seamounts, the western flank of the MAR, and the Great Meteor Seamounts exhibit geochemical anomalies [e.g., enriched (E-) MORBs, high concentrations of incompatible trace elements, and distinct isotopic ratios in oceanic crust], which are consistent with plume-ridge interaction (*7*, *14*). However, the geochemical affinity among these volcanic provinces is not straightforward. In particular, the New England Seamount samples exhibit higher ^208^Pb/^204^Pb at a given ^206^Pb/^204^Pb than the Azores lavas (*3*). Previous studies have argued that such differences may reflect spatial and temporal geochemical heterogeneity within a long-lived hotspot system (e.g., the Hawaii Loa-Kea trend and the Tristan-Gough hotspot track), indicating that mantle plumes can exhibit substantial differences, reflecting either source zonation within the plume conduit or changing sampling of mantle reservoirs through time (*35*–*37*). We therefore do not use these available geochemical data as definitive proof of a common plume source. Our models test whether a dynamically plausible connection could exist among the New England–Corner seamounts, the Great Meteor Seamounts, and the Azores seamounts. Further integrated isotopic and trace-element analyses are needed to resolve their detailed geochemical relationship, which is beyond the scope of this paper.

To conclude, we speculate that this absence of clear volcanic edifices can be explained by the rather inefficient, dispersed magmatism during ∼80 to 30 Ma. Similar irregular gaps in hotspot tracks have also been observed at other locations worldwide, such as the tracks generated by the Kerguelen and Reunion plumes (*38*–*40*). Both tracks involve multiple plates and passed through slow-spreading ridges during their formation. Consequently, we speculate that the plume-ridge interaction, subduction-induced mantle flow, and slow ridge’s spreading rate might be important factors that are closely related to the continuation of a straddle-plate hotspot track system.

### Influence of plate drag and sublithospheric mantle flow on the evolution of the plume

The combined effects of plate drag, ridge suction, and large-scale mantle flow have been proposed as key factors significantly affecting plume evolution. For the Hawaii-Emperor seamounts chain, plate drag and ridge suction are the dominant factors (*30*, *41*). When a ridge approaches, the plume can be attracted, and its conduit deflected toward it, resulting in the paleolatitude anomaly in the seamount chain (*30*). However, the case of the New England chain is another story, in which we propose that vigorous upper mantle flow induced by the Farallon slab subduction appears to be the first-order controlling factor. While the plume-ridge interaction is likely to play a secondary role (*41*).

Subduction-induced upper mantle flow is driven by pressure gradients, tending to flow from the higher-pressure zones to the lower ones (*41*–*44*). Since the Mesozoic, the western shore of North America has been continuously subducted, creating a massive amount of accumulated slab beneath the eastern shore (*44*–*48*). The surface topography of North America, as well as the flow in the deep upper mantle, was actively influenced by the ancient Farallon slab (*47*–*51*). On the basis of our numerical results (Figs. 4 and 5 and fig. S5), the significant horizontal pressure gradients on top of the upper mantle were created by the deepening and steepening of the retreating subducting Farallon slab, which caused vigorous long-distance horizontal suction and Poiseuille-type flow of the upper mantle toward the subduction trench. This Poiseuille-type flow mainly develops in the asthenospheric mantle, whereas it is much weaker near the imposed plume source depth of ∼600 km. Therefore, the westward flow should not be interpreted as directly determining the motion of the plume source at this depth. In addition, when the plume is located beneath the NAP, its conduit deviates westward, and the elongated axis of the quasielliptical plume head also points to the west rather than northwest with the motion of the plate (Figs. 3E, 5, A and G, and 6A). This hints that a trenchward, long-distance Poiseuille-type flow activated by continuous Farallon slab subduction exerts a stronger control on the deviation of the plume conduit and the morphology of the plume head than the drag from the NAP (Fig. 5 and fig. S5). Later, although the plume migrates to the other side of the MAR to the AFP, the deviation of plume conduit varies slightly. In contrast, the plume head is distinctly stretched and gradually evolves into an elongated, strip-like shape (Fig. 5, B to D and G to J). The mechanism behind this may be attributed to the increased drag originating from the superposition of sublithospheric mantle flow and the NAP motion, as the NAP has already shifted to move in nearly the same direction as the upper mantle flow (Fig. 5, C and D, and fig. S5). As a result, the plume head changes into an asymmetric, water-drop–shaped form, with its sharp corner still pointing west (Fig. 5, E, F, K, L, Q, and R). This morphological change likely reflects the slowdown of both the NAP movement and the sublithospheric mantle flow, the latter caused by the break-off of the ancient Farallon slab, which leads to a pressure gradient reduction (Figs. 3E and 5, K and L).

**Fig. 6.**
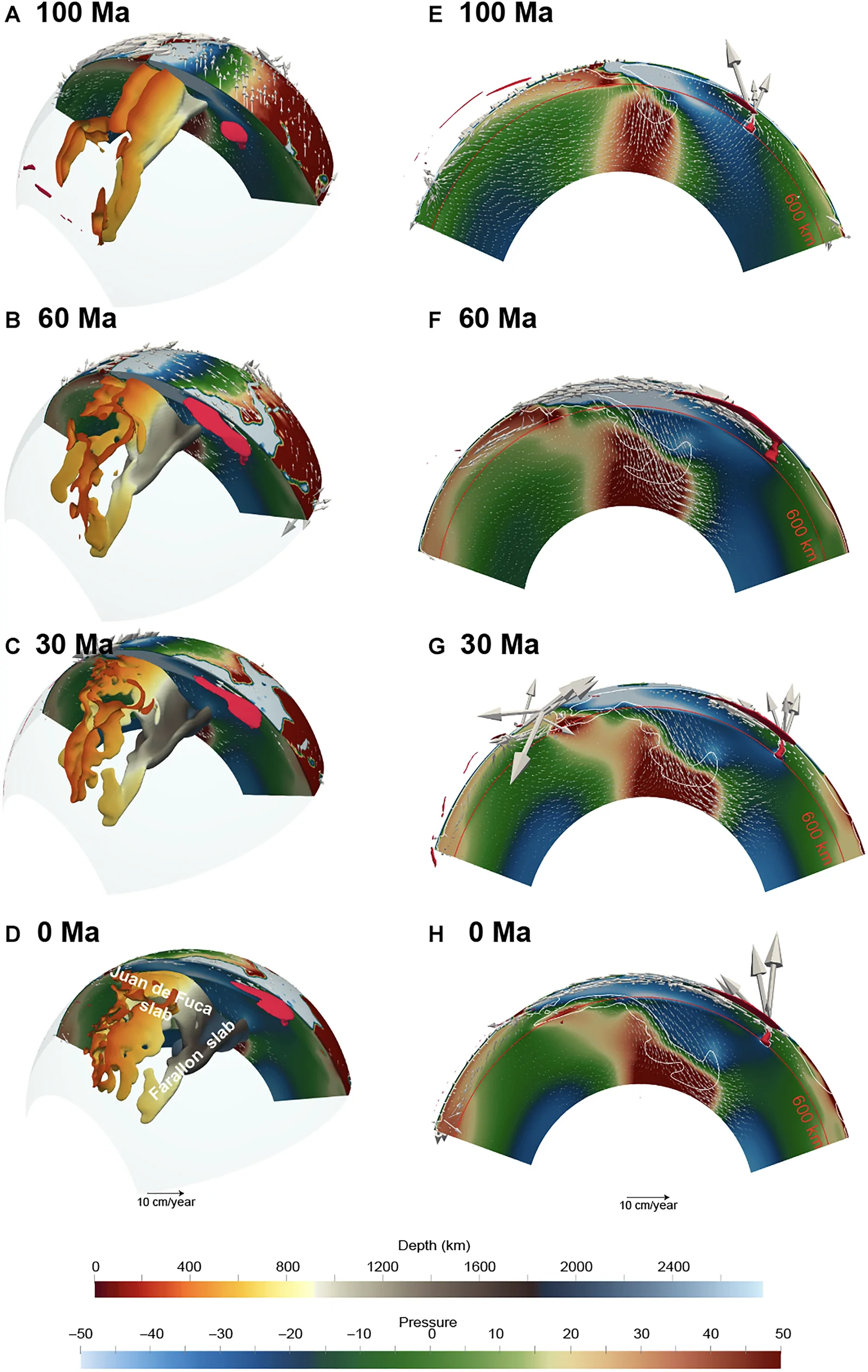
Evolution of pressure and velocity fields in model 3B. (**A**) 100 Ma, (**B**) 50 Ma, (**C**) 30 Ma, (**D**) 0 Ma. Slabs are outlined by temperature isosurface denoted by depth. (**E** to **H**) are the corresponding profiles showing the pressure fields. Slabs are outlined by white lines. The red lines are 600-km-depth contours. Gray arrows show the velocity, and their relative magnitude can be identified by the size. The plume in red is outlined by +100°C temperature anomaly.

To conclude, we propose that Poiseuille-type flow induced by Farallon slab subduction is the dominant factor exerting the first-order control on shaping the evolutionary path of the Azores plume. Plume-ridge interaction and plate motion provide secondary, superimposed contributions. Our model results further suggested that the influence of long-distance Poiseuille-type sublithospheric mantle flow extends at least as far as the MAR and is a far more significant driver of mantle dynamics beneath the North Atlantic Ocean than previously appreciated (Fig. 6).

In this study, we raised a new hypothesis that the Azores plume is the source of the New England Seamounts chain, supported by numerical simulations that show unprecedented agreement with the geological and geophysical observations than ever. Our model results indicate that the New England Seamounts chain and the Corner Seamounts chain on the NAP, the Great Meteor Seamounts on the AFP, and the Azores seamounts on the Eurasian Plate were all created by the same plume, moving in two phases and is currently located beneath the Azores seamounts. The primary factor, driving the plume’s morphology and the seamount chain evolution, is the long-distance Poiseuille-type flow within the sublithospheric mantle, caused by the deepening and steepening of the subducting Farallon slab. While plate motion and plume-ridge interaction show a secondary effect. Moreover, plume-plate interaction and ridge’s spreading rate are two important factors involved to the continuation of the trans-plate hotspot system.

## MATERIALS AND METHODS

### Model setup

To reproduce the history of the New England Seamounts chain while considering possible dynamic effects from all nearby plate boundaries, our regional models manage to include the subduction of the Farallon plate since the Late Mesozoic. Because parts of the subduction zone are too close to the North Pole, we conducted a coordinate transformation to improve the mesh of a regional model; the transformed computation domain is shown in fig. S1. Similarly, all the boundary data from the adopted plate reconstruction models are transformed as well.

We model the mantle as an incompressible viscous fluid under the Boussinesq approximation. Besides, we use a data assimilation method (*4*, *52*) to reproduce past subduction and mantle flow. In our model, all model boundaries, except the surface, are free to slip. The model’s surface assimilates kinematic boundary conditions from the plate reconstruction models, which also provide information about seafloor age and plate boundary geometries at a 1–million years (Myr) time interval (*33*, *48*). The input seafloor age and plate motion are smoothly interpolated between the two adjacent snapshots. The imposed plate motions are treated as kinematic constraints that drive mantle flow rather than being scaled to achieve dynamic self-consistency with a free-slip reference case.

In our models, the computational domain spans 120° in latitude and 210° in longitude. The study region is nearly in the center, and the plume does not move much laterally. Thus, this computational domain is large enough to capture the freely evolving subduction processes within the northeastern Pacific Ocean since the Early Cretaceous and to locate the plume far from the model boundaries. In addition, we also prescribe a thoroughly weakened vertical zone along all sidewalls to minimize the artificial flow due to the impermeable vertical boundaries (*50*, *53*). While prescribing the velocity field from global models as a boundary condition in regional models is an alternative approach to account for the far-field effect (*27*), it is crucial to ensure that the surface boundary condition from reconstructed plate motion is consistent with the velocity field modeled from buoyancy to prevent forced convection [e.g., (*54*–*56*)]. Previous studies (*55*) have proposed that regional model incorporating reconstructed plate motion as a surface boundary condition, as we did in this study, is appropriate for North American. Therefore, we believe the model setup here is reasonable. The model covers the whole mantle domain. It has a uniform radial resolution of ∼26 km, and the horizontal resolution varies from surface (∼52 km) to core-mantle boundary (∼29 km).

We solve for thermochemical convection, where compositional tracers at 20 per element are used to track key tectonic features, including the crust, the mantle, and the plume. Tracers with different physical properties (density and viscosity) are used to facilitate realistic subduction (*50*, *53*). Additional model components such as eclogite inside the plume and simplified melt migration are also realized through tracers.

The main difference between our model and previous studies (*17*) is that, instead of simply projecting the location of the plume conduit from its source to the surface, we further consider the lateral advection of the plume within the asthenosphere due to interaction with the moving plate at the surface. In addition, we also incorporate a simplified melting function to the evolution of the thermal plume, such that the melt could accumulate over time and advect with the ambient mantle flow. The plume conduit could then be bent by the lithosphere-plume interaction (*30*), while the melt region could move laterally within the plume head. As this method is more feasible for simulating melt eruption in oceanic lithosphere rather than the thicker continental lithosphere, all our models are run for ∼100 Ma to focus on the evolution of the tracks in the ocean.

For the purpose of testing the fixity of a plume, we construct two different kinds of models. In the first, the plume source is fixed at 600-km depth, assuming it is not significantly affected by background mantle flow. In the second, the plume source at the same depth is allowed to move at a rate of 0.1°/Ma (table S2), representing the scenario supposing that the plume stability is influenced by the mantle convection, including the subduction-induced flow, the thermal- and/or chemical-anomaly–induced flow, etc. The imposed plume motion is distinguished from potential plume motion at greater depths (e.g., near the core-mantle boundary), which is not explicitly resolved in our model. The difference between the mobility/stability of plumes near mantle transition zone and the core-mantle boundary may depend on plume intrinsic properties, on plate tectonics, on mantle flow, or on their proximity to mid-ocean ridges (*57*). Previous studies have suggested that the depth of the plume source would affect the evolution of plume (*58*, *59*). While the imposed plume parameters (e.g., plume excess temperature and radius) in our numerical models are chosen based on geophysical and geochemical observation constraints on upper-mantle plumes. Specially, the plume conduits are expected to be 100 to 200 km in diameter in the upper mantle (*2*, *15*, *23*, *60*) and the plume excess temperatures at ∼250° to 350°C or ∼19 to 27% of the ambient upper mantle temperature of 1280°C (*61*, *62*). Therefore, the model prediction could reproduce the first-order observed characteristics of the upper-mantle plume.

To track the formation of the New England Seamounts chain, we consider the melting process of the mantle (*63*–*65*). In this study, we only consider the melt of peridotite and adopt a linear melting function for the volumetric degree of melting (before considering extraction and depletion) φ_0_ (*53*)

$$\varphi_{0}=\text{min}\left[1,\text{max}\left(0,\frac{T-T_{solidus}}{T_{liquidus}-T_{solidus}}\right)\right]$$ (1)

where $T_{\text{solidus}}$ and $T_{\text{liquidus}}$ are pressure-dependent solidus and liquidus constrained by experiments on dry peridotite (*48*, *57*)

$$T_{\text{solidus}}=1080+134.2P–6.581P^{2}+0.1054P^{3}$$ (2)

$$T_{\text{liquidus}}=1762+57.46P–3.487P^{2}+0.077P^{3}$$ (3)

where *P* is pressure in gigapascals and *T* is temperature in °C. The pressure is calculated using a 1D reference density model (*44*).

We assume melt materials would erupt at a rate of 10%/Myr when the local melt percentage reaches a volumetric melt extraction threshold of $M_{\text{max}}=4\%$ (*30*). The value 10%/Myr means that when the local volumetric melt percentage reaches *M*_Max_, 10% of the locally available melt would erupt during 1 Myr. When an eruption happens, the erupted melt is put on the surface instantaneously. This simplified modeling may result in the depth at which the melt reaches the eruption threshold being very shallow, usually right below the Lithosphere-Asthenosphere Boundary (LAB). This means melt percolation happens mostly within the high-viscosity lithosphere, where the mantle wind is absent. Thus, this simplification would not cause a notable mismatch at the surface eruption site. Melt depletion $\varphi_{\text{extracted}}$ is also calculated to record the amount of erupted melt. The in situ melt fraction is $\varphi =\varphi_{0}-\varphi_{\text{extracted}}$.

### Model rheology

Our models adopt a depth-, temperature-, and composition-dependent viscosity law for the whole model domain before considering melting effects (*30*)

$$\eta_{\text{no}\;\text{melt}}=N_{0}\left(r\right)\cdot C\cdot \text{exp}\left[\frac{E_{0}\left(r\right)}{T+T_{0}\left(r\right)}-\frac{E_{0}\left(r\right)}{T_{m}+T_{0}\left(r\right)}\right]$$ (4)

where $\eta_{\text{no}\;\text{melt}}$ is the effective viscosity before considering melting effects, $N_{0}\left(r\right)$ is the depth-dependent background viscosity, *C* is the viscosity multiplier due to compositional effects, $E_{0}\left(r\right)$ is the activation energy, $T_{0}\left(r\right)$ is the temperature offset, and *r* is the radial distance from the earth’s core. *T* is temperature, and $T_{m}$ is the background temperature. These parameters and their values are listed in table S1. Most parameters have been used and tested in other studies (*24*, *30*).

The background mantle includes a four-layer viscosity profile: 0 to 44, 44 to 410, 410 to 660, and 660 to 2867 km. The background viscosity in each depth range is 10^20^, 10^20^, 10^21^, and 10^23^ Pa·s, respectively. The temperature dependence leads to four orders of magnitude in lateral viscosity variation. This viscosity structure is largely consistent with our previous inferences based on data assimilation models for simulating past subduction and mantle flow (*24*, *30*), which provide a tight range on the values of viscosity at each mantle depth. Besides, a viscosity cutoff (10^19^ to 10^23^ Pa·s) was used in our models to avoid too large a variation due to compositional and melting effects. These model parameters have been extensively tested in recent studies and shown to be appropriate for simulating subduction and mantle flow dynamics (*24*, *30*).

We consider two kinds of melt weakening mechanisms to account for melt effects on viscosity. The first concerns melt extraction, during which the viscosity of the whole rock column between the melt source and the surface would be weakened (*18*)

$$\eta =\zeta_{1}\cdot \eta_{\text{no}\;\text{melt}}$$ (5)

where $\zeta_{1}$ is the melt weakening prefactor. This mimics the process of melt eruption. The whole eruption channel from the node where melt is extracted to the surface would get weakened, following a recent study (*25*).

Another weakening mechanism arises from the fact that rheologically weak melt can be retained inside the mantle (*4*). We assume the effective viscosity depends on the in situ melt fraction following

$$\eta =\eta_{\text{no}\;\text{melt}}\times \text{exp}\left(-\zeta_{2}\times \varphi \right)$$ (6)

where $\eta_{\text{no}\;\text{melt}}$ and η are the effective viscosity before and after considering melting effects, respectively. $\zeta_{2}$ is another melt-weakening prefactor (*66*), and φ is the in situ melt fraction. Because the maximum in situ melt fraction in our model is limited to 4%, this weakening mechanism leads to a maximum viscosity reduction of one order of magnitude.

### Volcano tracking

Two different approaches are applied to get surface hotspot tracks (fig. S5): #A denotes predicted tracks from models with the vertical projection of the plume source onto the moving surface, and #B shows the model results incorporating the temporally changing location of the simulated melt eruption (# is the model number). These volcanic-tracking algorithms are designed to record melt occurrence and the erupted age rather than to evaluate whether the melt volume is sufficient to construct a preserved seamount edifice.

For track A, as we have prescribed the location of plume source at 600 km in the model, this location is then vertically projected onto the earth surface. We simply advect the corresponding tracers at the Earth’s surface following plate motions from the reconstruction models. For track B, after the erupted melt is moved to the surface instantly, it would be attached to an existing tracer near the surface, representing the volcano. Thus, it could move with the plates and even subduct under another plate. Because this melt has been moved to the surface and solidified, it has no effect on viscosity anymore. In practice, this volcano material is removed from the model when it subducts to a depth greater than 1000 km. As all tracks of #B show better predictions than the corresponding tracks #A (Figs. 2 and 4). In addition, according to model 3B (Fig. 5), significant lateral flow component, especially subduction-induced mantle flow, exists in the upper mantle. Thus, track #A neglecting such lateral flow is not suitable for tracking the volcanism.
