# Supplementary material for: The Azores plume origin of the New England Seamounts chain
Source: Sci Adv. 2026 Nov 13;12(46):eaee1780. doi: 10.1126/sciadv.aee1780 (PMC13763836; doi:10.1126/sciadv.aee1780)
Supplement: Supplementary file 1 [file sciadv.aee1780_sm.pdf]

Science Advances
AAAS

Supplementary Materials for

The Azores plume origin of the New England Seamounts chain

Mengxue Liu *et al.*

Corresponding author: Dinghui Yang, ydh@mail.tsinghua.edu.cn; Lijun Liu, ljliu@mail.iggcas.ac.cn

Sci. Adv. **12**, eaee1780 (2026)
DOI: 10.1126/sciadv.aee1780

This PDF file includes:

Figs. S1 to S5
Tables S1 and S2

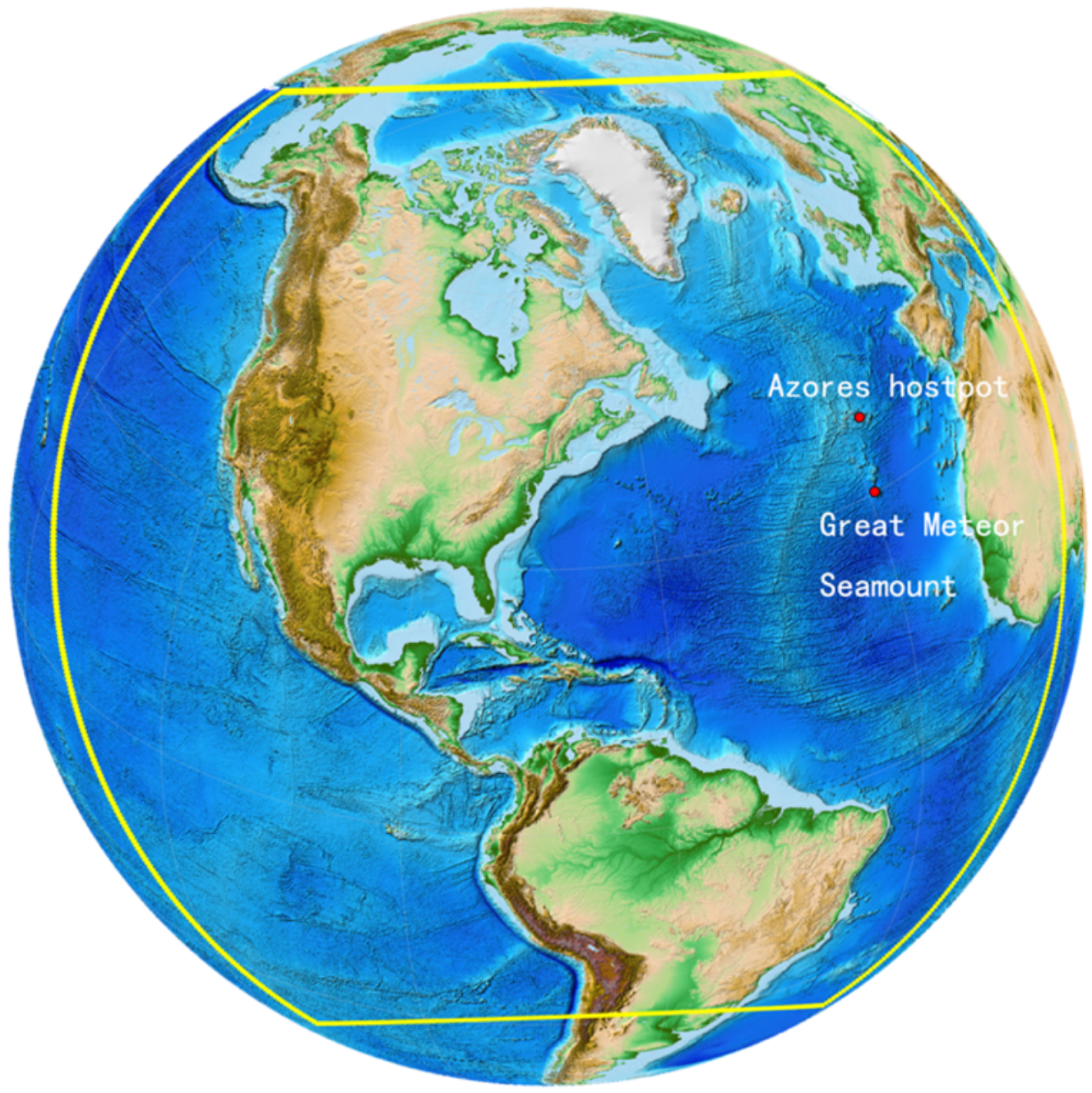

Fig. S1 The model computational domain. The lateral extents of our regional model are within the yellow outline, and the vertical extent is from the CMB to the surface.

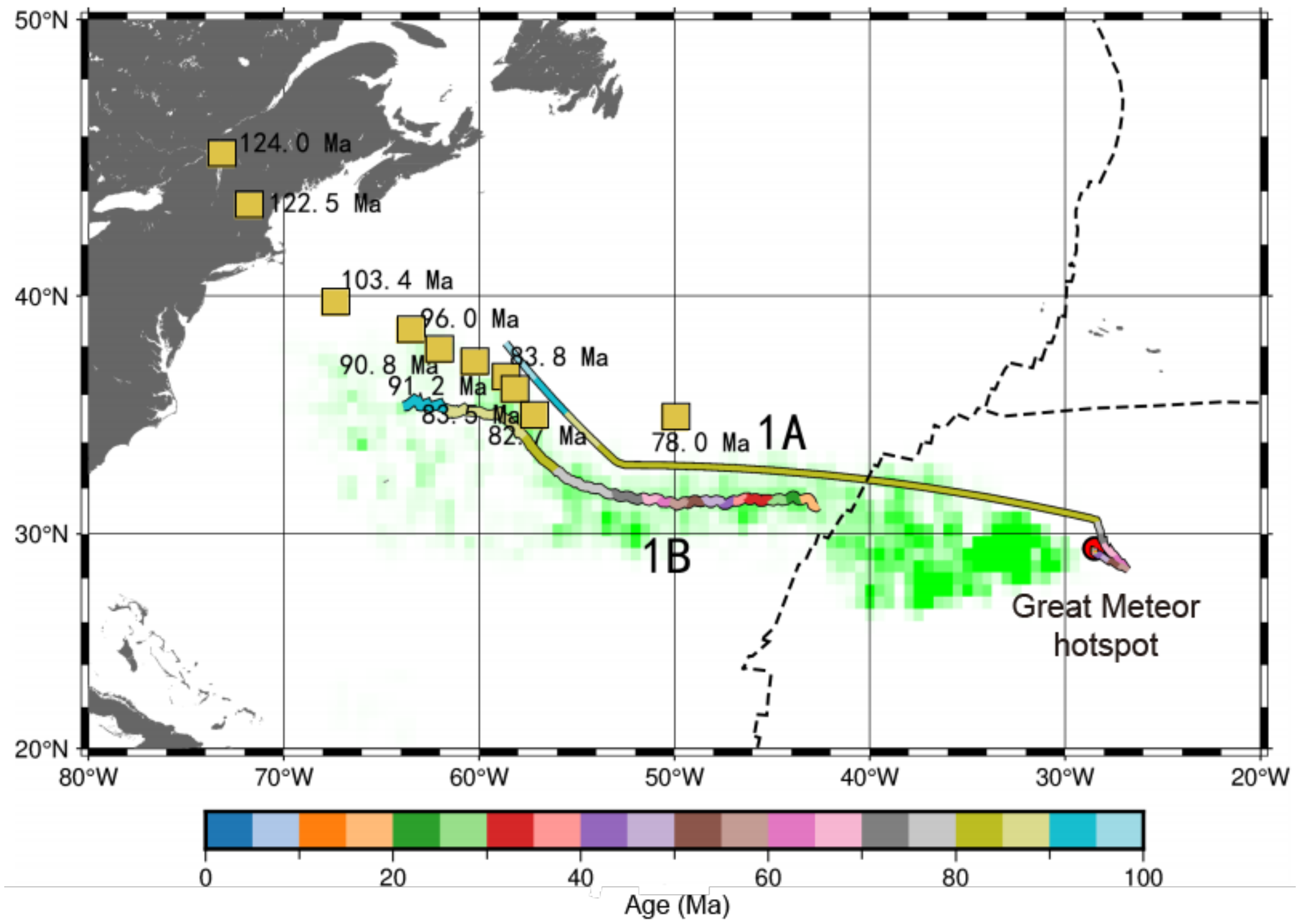

Fig. S2 Predicted hotspot tracks assuming the Great Meteor plume as the plume origin. Track 1A records the vertical projection of the plume source at ~600 km depth onto the moving surface, and track 1B records the temporally changing location of the simulated melt eruption. The green shades are sites of erupted melt predicted by Model 3B.

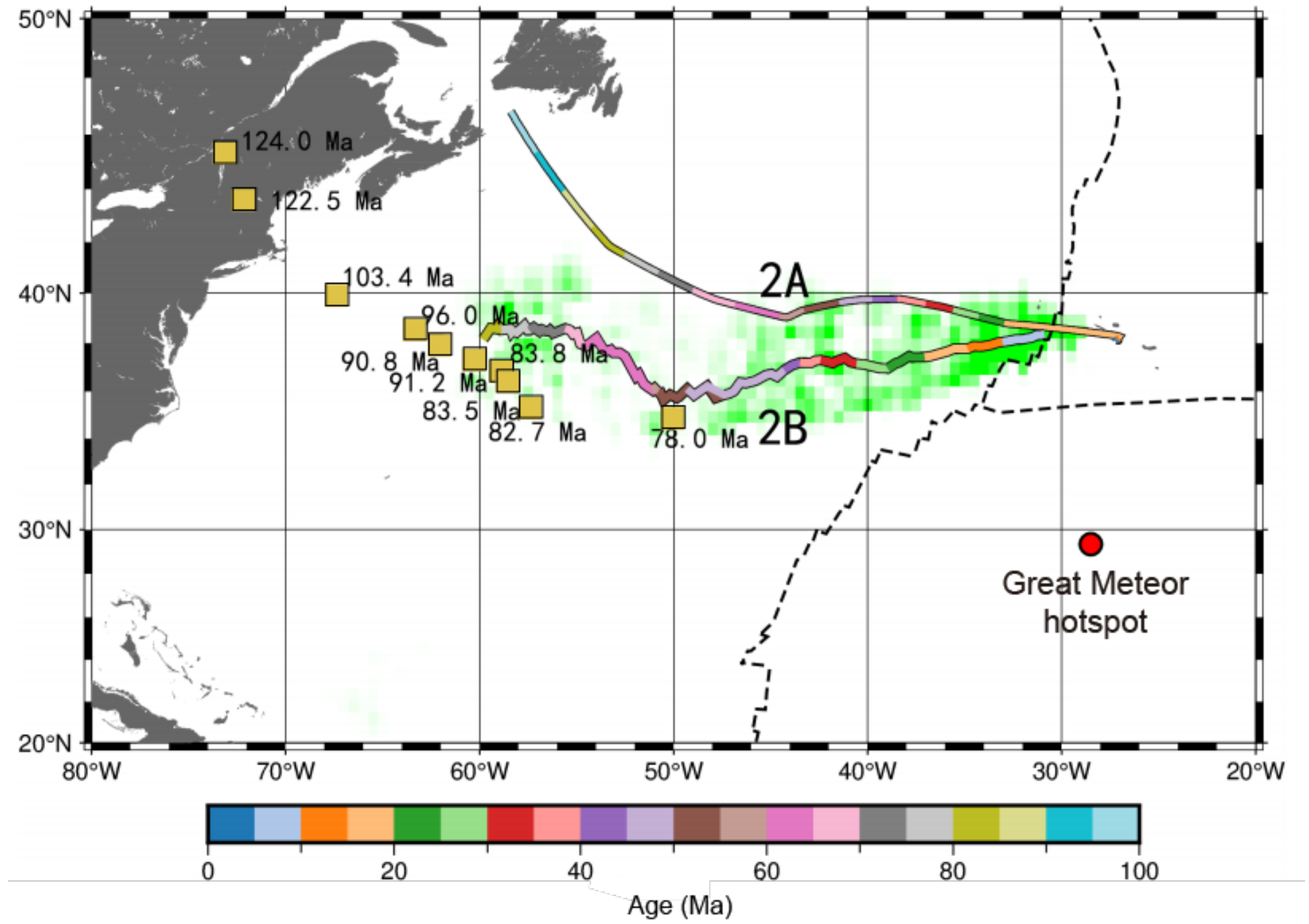

Fig. S3 Predicted hotspot tracks assuming the fixed Azores plume as the plume origin. Track 2A records the vertical projection of the plume source at ~600 km depth onto the moving surface, and track 2B records the temporally changing location of the simulated melt eruption. The green shades are sites of erupted melt predicted by Model 2B.

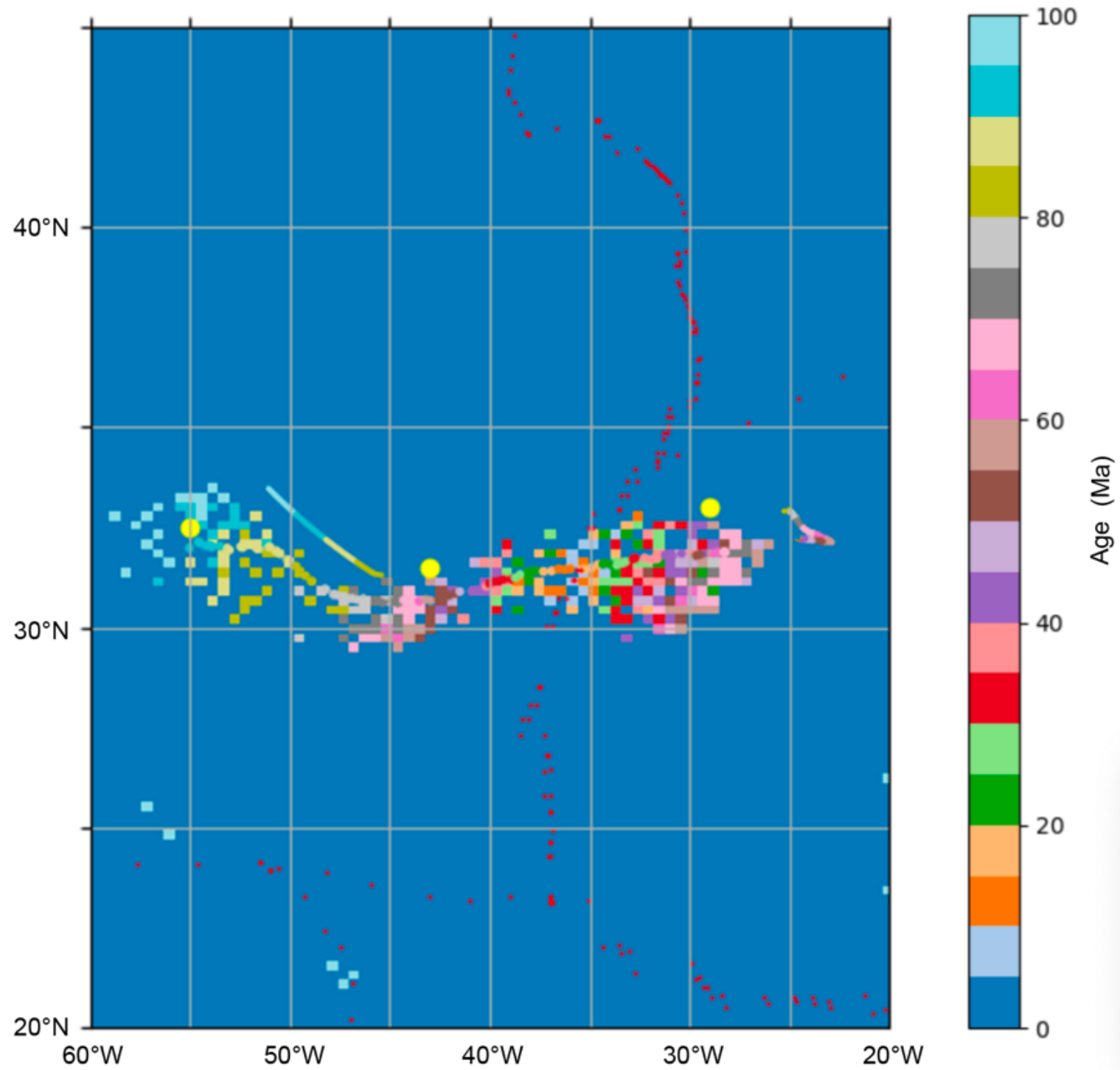

Fig. S4 Melts erupt sites (colored squares) in Model 3B, with the color on showing the eruption age. The small red dots outline the plate boundary.

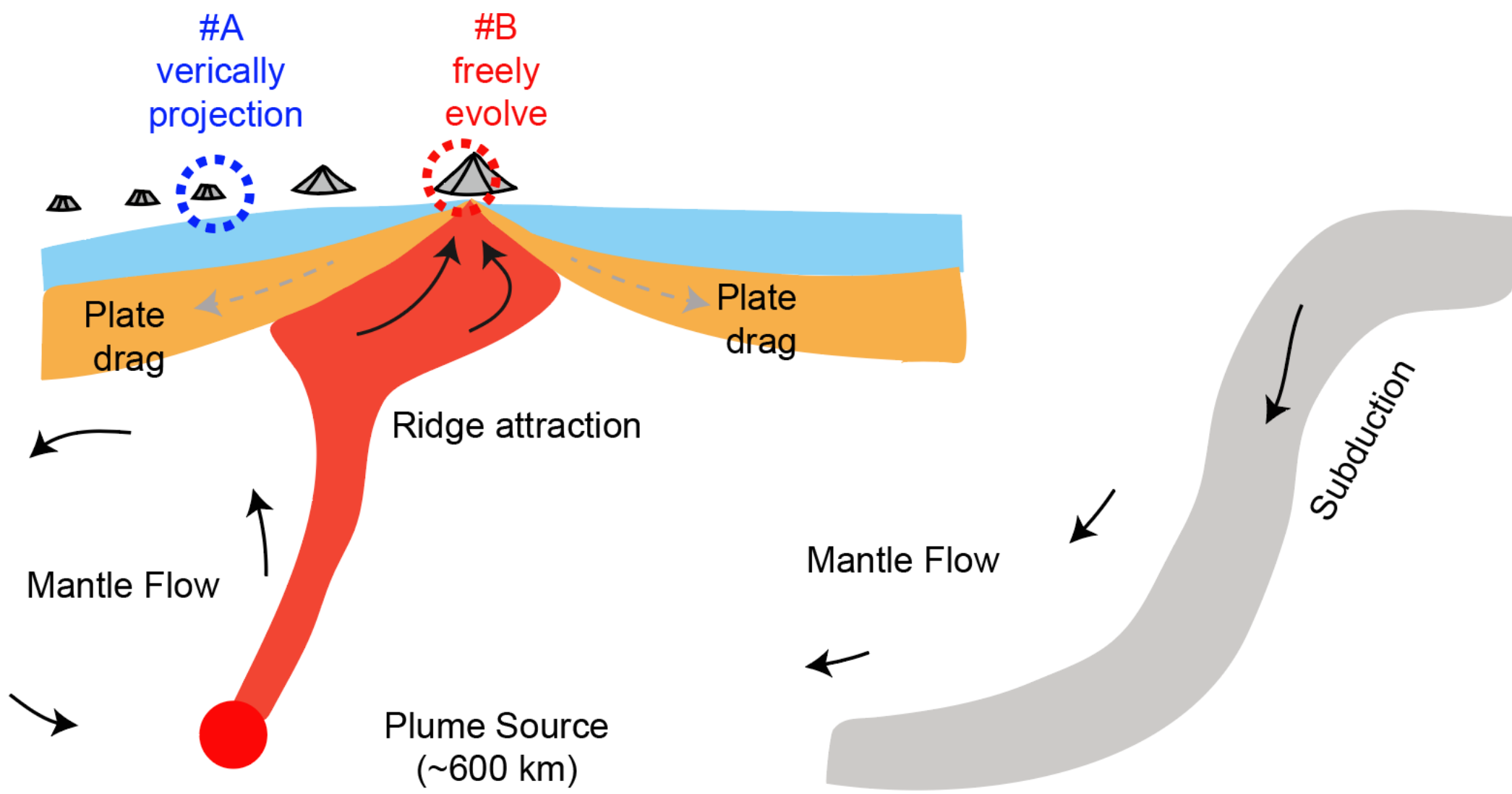

Fig. S5 Schematic sketch on how #A and #B methods of volcanic tracking work. #A denotes predicted tracks from models with the vertical projection of the plume source at ~600 km depth onto the moving surface and #B shows the model results incorporating the temporally changing location of the simulated melt eruption (# is the model number).

Table S1. Basic model parameters.

Parameter	Value	Nondimensional value
Earth's radius, R	6371 km	1
Mantle thickness, h	2867 km	0.45
Mantle density, ρ_m	3340 kg/m^3	1
Gravitational acceleration, g	9.8 m/s^2	-
Thermal diffusivity, κ	10^{-6} m^2/s	-
Thermal expansivity, α	3×10^{-5} K^{-1}	-
Mantle temperature[a], T_m	700 ºC	0.7
Reference viscosity, η_0	10^{21} Pa·s	1
Rayleigh number, Ra	2.6×10^8	-
Background temperature, T_m	700 ºC	0.7
Dynamic pressure, P	$\pm$120 MPa	$\pm$ 50
Activation energy, $E_0(r)$	25, 42, 25, 17 kJ/mol	3, 5, 3, 2
Temperature offset, $T_0(r)$	100, 100, 300, 300 ˚C	0.1, 0.1, 0.3, 0.3
Viscosity multiplier[b], C_0 *to* C_8	-	1, 10^{-6}, 100, 600, 300, 800, 300, 150, 1
Maximum viscosity cutoff, η_{max}	10^{23} Pa·s	100
Minimum viscosity cutoff, η_{min}	10^{19} Pa·s	0.01
Clapeyron slope at 410 km	4.0 MPa/K	0.019
Clapeyron slope at 660 km	-2.0 MPa/K	-0.01
Viscosity strengthening by melt extraction, ζ_1	0.1	-
Viscosity weakening by melt presence, ζ_2	11.5	-

[a] The background mantle temperature T_m=0.7 should not be interpreted as an ambient mantle temperature of 700 ˚C. It is a nondimensional reference value used in our numerical formulation (56).

[b] C_0 to C_8 denote mantle material, oceanic crust, eclogite, upper continental crust, lower continental crust, upper continental lithospheric mantle, middle continental lithospheric mantle, lower continental lithospheric mantle, and arc material, respectively.

Table S2. Model list.

Model	Plume Name	Plume fixity	Plume source/Ending Point	Plume source motion	Middle Point	Starting Point	Figure
1	Great Meteor	Fixed	29.34˚N, 28.50˚W	-		-	Fig. S2
2	Azores	Fixed	38.1˚N, 27.2˚W	-		-	Fig. S3
3	Azores	Move	38.1˚N, 27.2˚W	northeastward -> northwestward, 0.1˚/Myr	31.1˚N, 26.0˚W	28.1˚N, 31.2˚W	Fig. 2
4	Azores	Move	38.1˚N, 27.2˚W	due northward, 0.1˚/Myr	-	28.1˚N, 27.2˚W	Figs. 4A&4E
5	Azores	Move	38.1˚N, 27.2˚W	northeastward, 0.1˚/Myr	-	28.1˚N, 31.2˚W	Figs. 4B&4F
6	Azores	Move	38.1˚N, 27.2˚W	northwestward, 0.1˚/Myr	-	28.1˚N, 26.2˚W	Figs. 4C&4G
7	Azores	Move	38.1˚N, 27.2˚W	northeastward -> northeastward, 0.1˚/Myr	31.0 ˚N, 30.0˚W	28.1˚N, 31.2˚W	Figs. 4D&4H
